# Flu Hunter: Unlocking the Secrets of a Virus

**DOI:** 10.3201/eid2510.190880

**Published:** 2019-10

**Authors:** Sameh W. Boktor, Stephen Ostroff

**Affiliations:** Pennsylvania Department of Health, Harrisburg, Pennsylvania, USA (S.W. Boktor);; S Ostroff Consulting, Harrisburg (S. Ostroff)

**Keywords:** 1918 pandemic, avian flu, flu, gain of function research, human-animal interface, influenza, One Health, pandemic flu, Robert Webster, swine flu, virology, viruses, zoonoses

*Flu Hunter: Unlocking the Secrets of a Virus* offers an engaging and highly readable homage to the influenza virus by one of the world’s preeminent influenza virologists, Dr. Rob Webster (Figure). Although the book is nominally an autobiographical account of Webster’s career, the central figure is often the influenza virus itself, with each of the 17 chapters focusing on an influenza-related event from the past century. Drawing on experience from 50 years as a virologist, Webster provides a first-person account of what happened in each event, and why and how it happened. 

**Figure:**
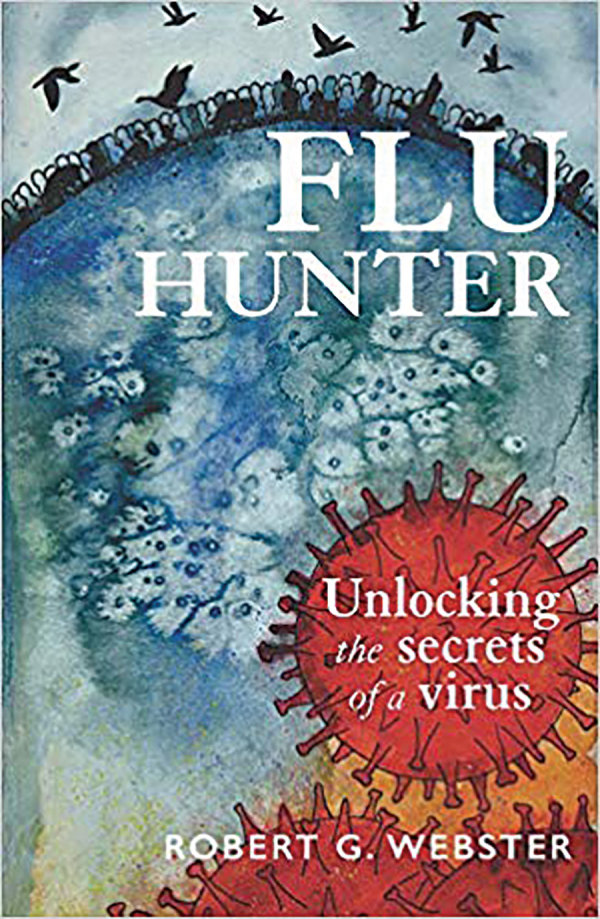
Flu Hunter: Unlocking the Secrets of a Virus

Webster points out that it was the heavy toll in human suffering caused by the devastating 1918 Spanish flu pandemic that jump-started influenza research and ultimately led the international scientific community to implement many clinical and public health improvements. Research from this and subsequent influenza events, including the 2009 H1N1 “swine flu” pandemic, has shaped our current understanding of influenza as a continuously mutating pathogen that demands constant global attention. 

Two themes appear throughout the book. The first is that influenza is a quintessential One Health pathogen that can only be understood when studied in humans and other animals but especially in both its wild and domestic bird reservoirs. Throughout his career, Webster has championed this approach, which has led to many of the breakthroughs in how we approach influenza prevention and control today. The second theme is the need for collaboration and cooperation to successfully address the challenges posed by influenza, an idea Webster has lived out by mentoring and working with influenza scientists throughout the world. 

Webster weaves anecdotes throughout the book about himself, his family, and his colleagues, among these a walk on an Australian beach littered with dead birds that led to his lifelong interest in influenza. There are wonderful descriptions of his involvement in field expeditions to the Great Barrier Reef, horseshoe crab nesting sites on Delaware Bay, and Spitsbergen, Norway. However, some sections of the book focus so intently on describing influenza viruses or the work of other investigators that readers may be left wanting to hear more of the remarkable personal stories he has to tell. 

The book does not entirely shy away from controversial topics. In the latter sections of the book, Webster addresses the pros and cons, from scientific and societal perspectives, of reconstituting the 1918 influenza virus. This section includes a discussion of gain of function research into the potential transmissibility of novel influenza viruses—research that might be used for the benefit or to the detriment of humankind. Webster concludes the book with a discussion of as-yet unanswered questions about the virus and how prepared the world is for an inevitable future pandemic. 

*Flu Hunter: Unlocking the Secrets of a Virus* offers a welcome addition to the bookshelf of anyone wanting to know more about the science of influenza, whether as an interested observer or a seasoned virologist. Indeed, science aside, this story of a remarkable, rewarding, and impactful career and life makes for compelling reading on its own. 

